# Use of Botanical Ingredients Relevant to Premenstrual Symptoms and Dysmenorrhea Among Elite Female Athletes: Evidence from Doping Control Forms

**DOI:** 10.3390/nu18203455

**Published:** 2026-10-21

**Authors:** Dominika Granda, Andrzej Pokrywka, Maria Karolina Szmidt, Olga Surała, Karol Jędrejko, Sylwia Lewandowska-Pachecka, Grażyna Nowicka

**Affiliations:** 1 Department of Nutrition Physiology, Institute of Sport— National Research Institute, 01-982 Warsaw, Poland; olga.surala@insp.pl; 2 Department of Biochemistry and Pharmacogenomics, Faculty of Pharmacy, Medical University of Warsaw, 02-097 Warsaw, Poland; andrzej.pokrywka@wum.edu.pl (A.P.); sylwia.lewandowska-pachecka@wum.edu.pl (S.L.-P.); grazyna.nowicka@wum.edu.pl (G.N.); 3 Scientific Team, Polish Anti-Doping Agency, 00-446 Warsaw, Poland; karol.jedrejko@gmail.com; 4 Department of Human Nutrition and Health Education, Faculty of Health Sciences, Warsaw University of Life Sciences (WULS-SGGW), 02-787 Warsaw, Poland; maria_szmidt@sggw.edu.pl

**Keywords:** dietary supplements, herbs, botanicals, premenstrual syndrome, dysmenorrhea, female athletes, menstrual cycle, doping control

## Abstract

Background/Objectives: Premenstrual syndrome (PMS) and dysmenorrhea can impair training performance in female athletes. Botanical ingredients are commonly used to manage these conditions in the general population, but it is unknown whether elite female athletes do so. This study aimed to identify botanical ingredients evaluated in studies for PMS and dysmenorrhea and determine whether elite female athletes declared them on Doping Control Forms (DCFs). Methods: A narrative literature review was conducted to identify botanical ingredients evaluated in studies for PMS and dysmenorrhea. Then, DCFs completed during routine anti-doping testing among elite athletes in Poland (2018–2023) were analyzed for declarations of these ingredients, both directly and as components of multi-ingredient products. The proportion of DCFs containing these ingredients was compared between female (*n* = 4451 DCFs) and male athletes (*n* = 9068 DCFs; reference) using chi-squared tests. Results: The review identified 8 and 21 botanical ingredients with evidence for PMS and dysmenorrhea, respectively; of which 9 were detected in DCFs. The most commonly declared ingredients among female athletes were ginger and turmeric/curcuminoid (1.75% and 1.26%, respectively, both studied for PMS and dysmenorrhea). Chasteberry, on the other hand, with the most extensive, albeit low-confidence evidence, for PMS, was rarely declared (0.22%). Direct declaration of ginkgo, valerian, evening primrose oil, and chasteberry was significantly more common among female than male athletes (0.22% vs. 0.02%, 0.36% vs. 0.03%, 0.47% vs. 0.03%, 0.22% vs. 0.02%; adjusted *p* < 0.05). Conclusions: Declarations of botanical ingredients studied for PMS and dysmenorrhea were infrequent on DCFs completed by female athletes. Because DCFs do not record the reason for use, these data cannot establish whether declarations reflected treatment of menstrual symptoms; studies assessing indication, symptom status and motivation are needed.

## 1. Introduction

Female athletes experience a range of sex-specific health conditions that can affect both health and athletic performance. Two of the most prevalent menstrual cycle-related disorders are premenstrual syndrome (PMS) and dysmenorrhea, with reported prevalence rates of 25.7% [1] and 32.3% [2], respectively. PMS is characterized by a cluster of physical, emotional, and behavioural symptoms, such as mood disturbances, bloating, fatigue, and irritability, which occur in the luteal phase of the menstrual cycle and resolve with the onset of menstruation [3]. Dysmenorrhea, on the other hand, refers to painful uterine cramping that occurs during menstruation, and can range from mild discomfort to debilitating pain that interferes with daily functioning [4]. Beyond their impact on physical and psychological well-being, both conditions may impair recovery, reduce training availability and consistency, and negatively influence competition readiness [5]. Although these effects may appear transient, the cumulative impact of PMS alone is significant. Based on an estimation, female athletes may experience approximately 1237–1621 days of PMS symptoms over the course of an athletic career, equivalent to over three to four years [1]. Despite this, menstrual cycle-related disorders are frequently underestimated by athletes, coaches, and support staff alike, and consequently not properly addressed.

Dietary supplements are widely used among athletes to support health and address various health-related concerns [6]. In this context, products containing botanical ingredients may offer a potential strategy for managing PMS and dysmenorrhea [7,8]. Several botanicals have been clinically investigated for these conditions, with findings indicating potential benefits for symptom relief, although the strength and quality of evidence vary considerably across individual ingredients and clinical outcomes [7,8]. However, it is worth noting that none of these studies were conducted among elite athletes. The selection of botanical ingredients in elite sport requires careful consideration of scientific evidence, product quality, safety, and compliance with anti-doping regulations [9]. Dietary supplement use among athletes may carry adverse effects, including not only detrimental health consequences but also an increased risk of an adverse analytical finding, owing to the potential for contamination or adulteration, or the presence of botanically derived compounds whose pharmacological properties or anti-doping status may be unclear [10], potentially leading to inadvertent doping.

Whether female athletes use botanical ingredients for the management of PMS and dysmenorrhea, and the extent to which their choices align with current clinical evidence, has not previously been investigated. This knowledge gap is particularly important given the unique demands of elite sport. The aim of this study was to examine the frequency with which botanical ingredients evaluated in studies for the management of PMS and dysmenorrhea were declared on DCFs by elite female athletes. To address this aim, relevant botanical ingredients supported by evidence from clinical studies were first identified through a review of the literature and subsequently searched for in Doping Control Forms (DCFs) completed during routine anti-doping testing. Because DCFs do not record athletes’ reasons for supplement use, male athletes’ DCFs were included as an exploratory contextual comparison to help characterize patterns of declaration that may be plausibly linked to PMS/dysmenorrhea management.

## 2. Materials and Methods

### 2.1. Study Design

**Figure 1:**
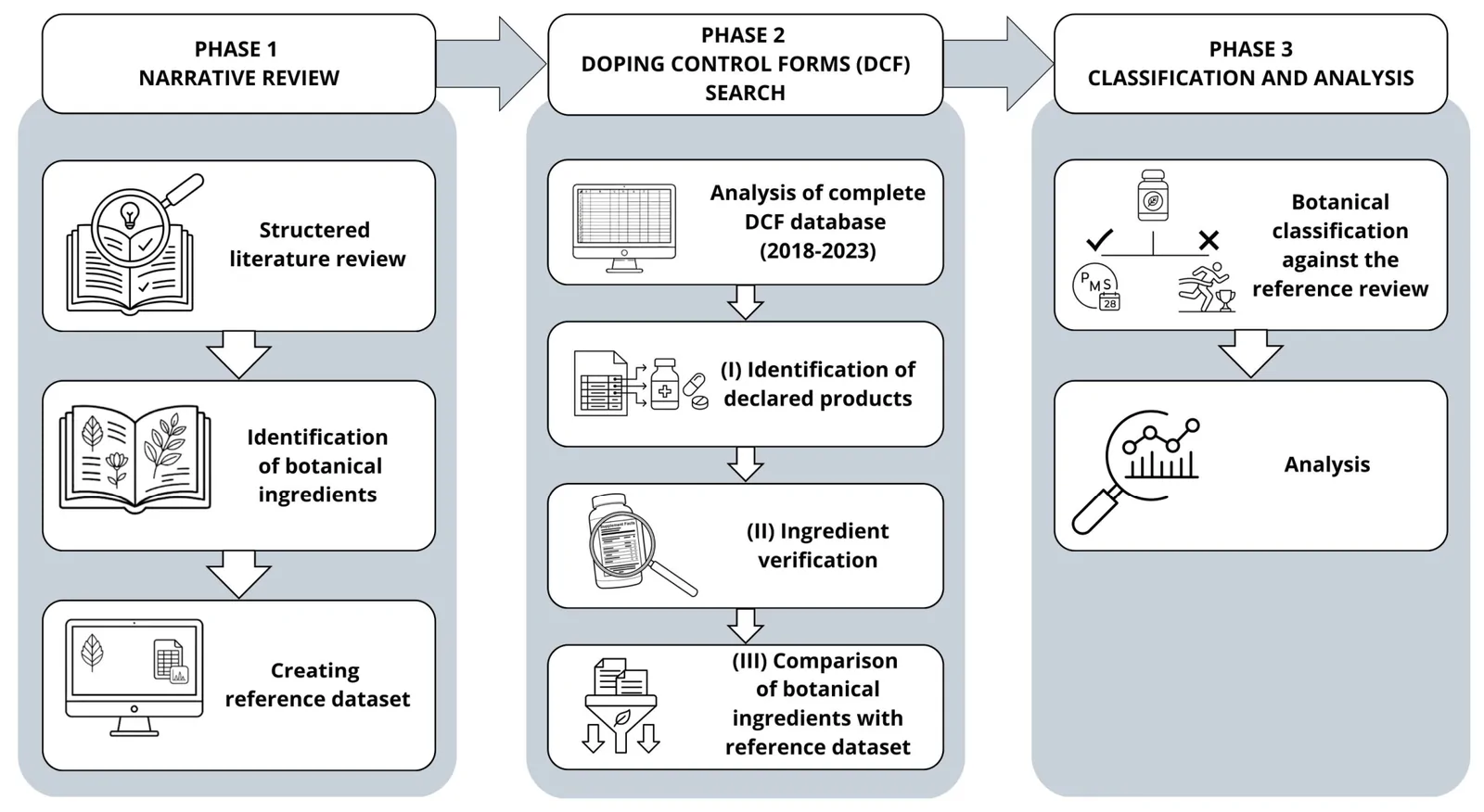
Overview of the study design.

The present study was conducted in three phases to bridge the gap between clinical evidence and practice regarding the use of botanical ingredients in the management of PMS and dysmenorrhea among elite athletes (Figure 1). Phase 1 consisted of a narrative literature review aimed at identifying botanical ingredients evaluated in studies for the management of PMS and dysmenorrhea. Phase 2 consisted of retrospective analysis of DCFs completed by athletes during routine doping controls carried out in Poland between 2018 and 2023, and Phase 3 involved an analysis to determine the frequency and patterns of declaration of the identified botanical ingredients on DCFs completed by elite Polish athletes.

The study was approved by the Ethics Committee of the Institute of Sport—National Research Institute (KEBN-23-79-OS, issued on 8 March 2023).

#### 2.1.1. Phase 1—Literature Review

The databases PubMed and Google Scholar were searched from inception to 16th August 2026 using combinations of keywords related to PMS, dysmenorrhea, botanical medicine, herbal medicine, phytotherapy, and dietary supplements (Phase 1, Figure 1); the full search strategies are provided in Supplementary Table S1. This was a narrative rather than a systematic review; no protocol was registered a priori, and formal systematic-review procedures (e.g., duplicate independent screening) were not applied. Eligible sources included clinical trials, interventional studies, systematic reviews, and meta-analyses written in English and published in peer-reviewed journals. We limited our review to evidence from human studies. In addition, we focused exclusively on botanical ingredients and did not include vitamins, minerals, or other micronutrients, despite the available evidence supporting their role in managing PMS and dysmenorrhea [11,12,13]. This decision was based on two considerations. Firstly, botanical ingredients used specifically for gynecological conditions remain underrepresented in the sports science literature, reflecting a research gap this study aimed to address. Secondly, declarations of vitamins and minerals on DCFs could not be reliably attributed to symptom management, as athletes commonly use such supplements for reasons unrelated to PMS or dysmenorrhea, including prevention of nutritional deficiencies and performance optimization. The botanical ingredients identified through this process were tabulated according to their common and Latin name.

The design-based level of evidence for each botanical ingredient for premenstrual symptoms and dysmenorrhea was assessed using the Oxford Centre for Evidence-Based Medicine (OCEBM) 2011 Levels of Evidence for questions of treatment benefit [14]. Under this framework, ingredients supported by at least one systematic review or meta-analysis of randomized controlled trials were classified as Level 1; while those for which only individual randomized controlled trials were available, in the absence of a dedicated systematic review, were classified as Level 2. As this analysis was restricted to evidence from human clinical trials, no supporting evidence for an ingredient fell below Level 2 (i.e., non-randomized controlled studies, case series, case–control studies, or mechanism-based reasoning). Consistent with OCEBM guidance, an ingredient otherwise meeting Level 1 criteria was graded down to Level 2 where methodological limitations warranted it, namely, if a systematic review’s conclusion was based on a single included trial rather than a pooled synthesis of multiple trials, or if the supporting trial evidence pertained to a multi-ingredient formulation rather than the botanical ingredient in isolation. Botanicals for which the only controlled evidence came from a multi-ingredient formulation, in which their independent effect could not be identified, were not included in the reference set. Evidence level reflects study design only and does not indicate certainty or magnitude of benefit. For each ingredient, we therefore also assessed the methodological quality of the cited systematic reviews, meta-analyses and randomized controlled trials, and report it alongside the evidence level in Table 1 and Table 2.

Systematic reviews and meta-analyses were assessed using A MeaSurement Tool to Assess systematic Reviews (AMSTAR 2) [15], a 16-item instrument for appraising systematic reviews of randomized and non-randomized studies of healthcare interventions. Seven items are considered critical domains: registration of a protocol before commencement of the review, adequacy of the literature search, justification for excluding individual studies, risk of bias assessment of included studies, appropriateness of meta-analytical methods, consideration of risk of bias when interpreting results, and assessment of publication bias. In line with AMSTAR 2 guidance, no summary score was calculated; instead, an overall confidence rating was assigned based on the number and type of weaknesses: high (no or one non-critical weakness), moderate (more than one non-critical weakness), low (one critical flaw, with or without non-critical weaknesses), or critically low (more than one critical flaw, with or without non-critical weaknesses). Item-level AMSTAR 2 judgements for each review are presented in Supplementary Table S2.

Randomized controlled trials were assessed using the revised Cochrane risk-of-bias tool (RoB 2) [16], which evaluates five domains: bias arising from the randomization process, bias due to deviations from intended interventions, bias due to missing outcome data, bias in measurement of the outcome, and bias in selection of the reported result. Each domain was judged as low risk, some concerns or high risk, and an overall risk-of-bias judgement was derived according to the RoB 2 algorithm: low if all domains were at low risk, some concerns if at least one domain raised some concerns but none was at high risk, and high if at least one domain was at high risk or several domains raised some concerns that substantially lowered confidence in the result. Each assessment was applied to a single prespecified result, defined by the outcome, time point and comparison assessed, with the effect of assignment to the intervention as the effect of interest. Crossover trials [17,18,19] were assessed using the RoB 2 variant for crossover trials, which includes an additional domain for bias arising from period and carryover effects [20]. The assessed result and domain-level judgements for each trial are presented in Supplementary Table S3. For each randomized controlled trial, the plant part and extract type, standardization, formulation, dose, duration and comparator were extracted and presented in Supplementary Table S4; where this information was not reported in the original publication, it is indicated as not reported (NR). Where Level 2 evidence was derived from a single trial included in a broader systematic review, the AMSTAR 2 rating of that review was reported. Assessments were performed independently by two co-authors (D.G., A.P.), with disagreements resolved by discussion.

#### 2.1.2. Phases 2 and 3—Doping Control Forms Analysis

DCFs completed by elite athletes across various sport disciplines during routine doping control tests carried out in Poland between 2018 and 2023 were obtained from the Polish Anti-Doping Agency (POLADA). DCFs are official documents completed at the time of doping control, in which athletes are required to declare all medications and supplements consumed in the seven days preceding the test. These declarations are recorded by trained Doping Control Officers. All forms were fully anonymized prior to analysis, in accordance with the study design applied in research performed previously on similar databases [21,22]. The available information included athlete sex, age, sport discipline and all medications and dietary supplements declared during the seven-day reporting period.

The identification and classification of botanical ingredients with potential benefits for PMS and dysmenorrhea followed a predefined multistep procedure designed to minimize the risk of errors arising from the various ways in which athletes reported products. Depending on individual reporting practices, declarations included either the commercial product names, active ingredients or only a general description of the product (e.g., “supplements provided by sport association”, or “herbal medicine”). Whenever a commercial product could be unequivocally identified, its complete composition was verified using manufacturers’ official websites used as the primary source whenever available. Additional publicly available sources, including online retailers, pharmacies, and supplement databases, were consulted when necessary to confirm product identity or composition. However, DCFs did not routinely contain sufficient information to establish the exact product version, manufacturing batch, or formulation used at the time of doping control. Consequently, the identification of botanical ingredients from commercial product names should be regarded as a retrospective reconstruction based on available product information rather than direct verification of the composition of the products actually consumed. Product declarations were processed and the database was updated regularly during the study period, beginning in 2018. The complete DCF database was then systematically screened using three consecutive steps: (i) identification of each declared product, (ii) verification of its full ingredient composition regardless of the product’s apparent indication, (iii) comparison of botanical ingredients against the predefined reference dataset of botanical ingredients supported by clinical evidence for PMS or dysmenorrhea, to determine their relevance to the present analysis. Thus, the final analysis included only botanical ingredients with clinical evidence supporting their potential benefits for PMS or dysmenorrhea symptom management, hereafter referred to as “relevant botanical ingredients”. Steps (i)–(iii) were performed independently by two investigators (A.P. and D.G.), and any discrepancies were discussed until consensus was reached (Phase 2, Figure 1). To ensure consistency, botanical ingredients reported under different commercial names or formulations were harmonized and analyzed according to their botanical identity rather than their individual product names.

For each botanical ingredient, the declarations on the DCFs were classified as either direct or indirect (Phase 3, Figure 1). Direct declarations were those in which the athlete explicitly listed the botanical ingredient (or its Latin/common name) as a substance or supplement taken. Indirect declarations were those in which the ingredient was not named by the athlete directly but was identified through the stated composition of a declared branded product. The total number of declarations per ingredient represents the sum of the direct and indirect declarations, by sex.

Information regarding the reasons for specific ingredient use was not available, as they are not recorded on standard DCF; thus, we included DCFs from both female and male athletes in the analysis. DCFs completed by male athletes were used as an exploratory contextual comparison to help characterize whether declaration patterns for these botanical ingredients were more consistent with the management of PMS or dysmenorrhea symptoms/female-specific indications or with non-specific, alternative purposes (e.g., adaptogenic, anti-inflammatory, or performance-enhancing properties), although DCF data cannot establish indication directly.

### 2.2. Statistical Analysis

The DCF served as the primary unit of analysis. Descriptive statistics and sex-based comparisons were used to analyze the data. Results were expressed as absolute frequencies and percentages, with the total number of DCFs serving as the denominator for all proportions; all percentages presented in this study are based on DCF counts unless otherwise specified. As all forms were fully anonymized, it was not possible to determine whether any athlete contributed more than one observation; independence between DCFs was therefore assumed but could not be verified. Differences in botanical ingredient declaration rates between male and female athletes were assessed using the chi-squared test. The distribution of the number of relevant ingredients per DCF was compared between sexes among DCFs containing at least one relevant ingredient (conditional analysis). Given the retrospective design of this study, the inability to determine indication for supplement use from DCF data, and the independence limitation noted above, all statistical comparisons in this study were treated as exploratory rather than confirmatory. To account for multiple testing across the ingredient-specific comparisons, *p*-values were additionally adjusted using the Benjamini–Hochberg false discovery rate procedure, applied to all 18 comparisons (nine botanical ingredients, each for total and direct declarations) as a single family of tests. As a sensitivity analysis, the procedure was also applied separately to the comparisons for total and for direct declarations (9 tests each); this did not change any conclusion regarding statistical significance. Effect sizes were expressed as both prevalence ratios (female:male) and absolute differences in declaration frequency, each with 95% confidence intervals. All calculations were performed using Statistica 13. Statistical significance was set at *p* < 0.05.

## 3. Results

### 3.1. Botanical Ingredients for Management of PMS Symptoms

Based on a review of the literature, we identified eight botanical ingredients that were evaluated for the management of premenstrual symptoms (Table 1). Three of these were classified as Level 1, while the remaining five were classified as Level 2.

**Table 1:** Botanical ingredients relevant for premenstrual syndrome management and their levels of clinical evidence.

Common Name	Latin Name	References	Quality Assessment
Level 1—systematic reviews and meta-analyses (AMSTAR 2)			
Chasteberry	*Vitex agnus-castus*	[23,24,25]	Low [23,24]; Critically low [25]
Saffron	*Crocus sativus*	[25,26]	Critically low [25,26]
Ginkgo	*Ginkgo biloba*	[25]	Critically low [25]
Level 2—single interventional studies (RoB 2)			
Evening primrose oil	*Oenothera biennis*	[27,28]	Some concerns [27]; High [28]
Turmeric/Curcuminoid ^1^	*Curcuma longa*	[29]	Some concerns
St. John’s wort	*Hypericum perforatum*	[17]	Low
Wild yam	*Dioscorea esculenta*	[18]	High
Ginger	*Zingiber officinale*	[30]	High

^1^ Includes clinical evidence for both whole turmeric extract and isolated curcuminoid preparations, given their shared botanical source (*Curcuma longa*).

Chasteberry (*Vitex agnus-castus*) fruit extract was identified as the supplement with the strongest evidence for alleviating PMS symptoms, as its efficacy was supported by two meta-analyses [23,24] and a systematic review [25]; however, confidence in all three reviews was rated as low or critically low, and these findings should therefore be interpreted with caution. Similar, but more limited evidence was found for saffron (*Crocus sativus*) [25,26]. Ginkgo (*Ginkgo biloba*) was assessed as more effective than a placebo in a systematic review by Dante and Facchinetti (2011) [25].

The beneficial effects of evening primrose oil (*Oenothera biennis*) were reported in two studies [27,28]. In one of them, participants received a single-ingredient supplement [28], whereas in the other study, evening primrose oil was administered as a part of a multi-ingredient supplement [27]. Turmeric/curcuminoid (*Curcuma longa*) supplementation was evaluated in a randomized, placebo-controlled trial using isolated curcumin as a stand-alone supplement [29]. An additional study evaluating a multi-ingredient supplement containing curcuminoids was identified but not included in Table 1, as it employed a single-arm, uncontrolled design without a comparator [31]. Reductions in PMS severity were reported for St. John’s wort (*Hypericum perforatum*) [17], wild yam (*Dioscorea esculenta*) [18] and ginger (*Zingiber officinale*) [30] in individual studies.

### 3.2. Botanical Ingredients for Management of Dysmenorrhea

Based on the literature review, we identified 21 botanical interventions evaluated for the management of dysmenorrhea, of which 11 were classified as Level 1 (including 7 individual botanical ingredients and 4 multi-botanical formulations) and the remaining 10 as Level 2 (Table 2).

**Table 2:** Botanical ingredients relevant for dysmenorrhea management and their levels of clinical evidence.

Common Name	Latin Name	References	Quality Assessment
Level 1—systematic reviews and meta-analyses (AMSTAR 2)			
Individual botanical ingredients			
Ginger	*Zingiber officinale*	[32,33,34]	Critically low [32,33]; Low [34]
Fennel	*Foeniculum vulgare*	[32,35]	Critically low [32,35]
Chamomile	*Matricaria chamomilla*	[34,36]	Low [34]; Critically low [36]
Damask rose	*Rosa damascena*	[37]	Low
Cinnamon	*Cinnamomum cassia*	[32]	Critically low
Saffron	*Crocus sativus*	[26]	Critically low
Dill	*Anethum graveolens*	[35]	Critically low
Multi-botanical formulations (traditional Chinese medicine)			
Wenjing Huoxue Decoction (WJHXD) ^1^	*Corydalis yanhusuo*, *Glycyrrhiza glabra*, *Commiphora myrrha*	[38]	Low
Shaofu Zhuyu decoction	*Decoctum Shaofu Zhuyu*	[39]	Critically low
Danggui Shaoyao San	*Decoctum Angelicae sinensis et Paeoniae albae*	[40]	Critically low
Xuefu Zhuyu decoction	*Decoctum Xuefu Zhuyu*	[41]	Low
Level 2—single interventional studies (AMSTAR 2/RoB2)			
Guava leaf	*Psidium guajava*	[34,42]	AMSTAR 2: Low [34]; RoB 2: High [42]
Rhubarb	*Rheum emodi*	[34,43]	AMSTAR 2: Low [34]; RoB 2: High [43]
Wild yam	*Dioscorea esculenta*	[18]	RoB 2: High
Chlorella	*Chlorella vulgaris*	[44]	RoB 2: Low
Turmeric/Curcuminoid ^2^	*Curcuma longa*	[45]	RoB 2: Some concerns
Fenugreek	*Trigonella foenum-graecum*	[34]	AMSTAR 2: Low
Valerian	*Valeriana officinalis*	[34]	AMSTAR 2: Low
Zataria	*Zataria multiflora*	[34]	AMSTAR 2: Low
Dried figs	*Ficus carica*	[46]	RoB 2: High
Stachys lavandulifolia	*Stachys lavandulifolia*	[19]	RoB 2: High

^1^ WJHXD = Wenjing Huoxue Decoction; a traditional Chinese medicine formula composed of multiple botanical components including Corydalis yanhusuo (Yan Hu Suo), Glycyrrhiza glabra (liquorice), and Commiphora myrrha (Myrrha/Mo Yao). ^2^ Includes clinical evidence for both whole turmeric extract and isolated curcuminoid preparations, given their shared botanical source (*Curcuma longa*).

The strongest evidence was found for ginger (*Zingiber officinale*), with its effectiveness supported by two meta-analyses [32,33] and a systematic review [34]. Fennel’s (*Foeniculum vulgare*) effectiveness was also reported in two meta-analyses [32,35], and chamomile’s (*Matricaria chamomilla*) in two systematic reviews [34,36]. A single meta-analysis supported the effectiveness of damask rose [37] (*Rosa damascena*; for secondary symptoms such as headache, fatigue and bloating, but not menstrual pain), cinnamon [32] (*Cinnamomum cassia*), saffron [26] (*Crocus sativus*) and dill [35] (*Anethum graveolens*). In addition, we identified four multi-botanical formulations commonly used in traditional Chinese medicine, namely Wenjing Huoxue Decoction (*Corydalis yanhusuo*, *Glycyrrhiza glabra*, *Commiphora myrrha*), Shaofu Zhuyu decoction (*Decoctum Shaofu Zhuyu*), Danggui Shaoyao San (*Decoctum Angelicae sinensis et Paeoniae albae*), and Xuefu Zhuyu decoction (*Decoctum Xuefu Zhuyu*). Their potential benefit was reported in single meta-analyses for Wenjing Huoxue Decoction [38], Danggui Shaoyao San [40], and Xuefu Zhuyu decoction [41], and in a systematic review for Shaofu Zhuyu decoction [39]. In all cases, however, the authors noted that this evidence should be considered suggestive given the high risk of bias identified in the included studies.

Among the Level 2 substances, evidence was generally limited to single studies and findings were not always consistent. Guava leaf (*Psidium guajava*) showed dose-dependent effects, with the higher dose (6 mg/day) significantly reducing pain compared with both placebo and ibuprofen in one trial [42], although evidence from a Cochrane review based on lower-dose comparisons was inconsistent [34]. Rhubarb (*Rheum emodi*) showed no significant difference compared with NSAIDs according to a systematic review [34], but significant within-group symptom improvement comparable to mefenamic acid in another [43]. Eight further substances—wild yam (*Dioscorea esculenta*) [18], chlorella (*Chlorella vulgaris*) [44], turmeric/curcuminoid (*Curcuma longa*) [45], fenugreek (*Trigonella foenum-graecum*) [34], valerian (*Valeriana officinalis*) [34], zataria (*Zataria multiflora*) [34], dried figs (*Ficus carica*) [46], and Stachys lavandulifolia (*Stachys lavandulifolia Vahl*) [19]—were classified as Level 2, with potential benefit reported in single interventional studies.

### 3.3. Quality Assessment

Of the 14 systematic reviews and meta-analyses assessed with AMSTAR 2, six were rated as low [23,24,34,37,38,41] and eight as critically low [25,26,32,33,35,36,39,40] confidence; none reached moderate or high confidence (Table 1 and Table 2). The most common critical weaknesses were inadequate assessment or discussion of publication bias [23,33,34,35,38,39,40,41] and the absence of an a priori registered protocol [24,25,26,32,36]. Of the 12 randomized controlled trials assessed with RoB 2, two were rated as low risk of bias (St. John’s wort [17] and chlorella [44]), three as some concerns [27,29,45], and seven as high risk of bias [18,19,28,30,42,43,46], most frequently due to concerns in the randomization process and in the selection of the reported result. Standardization or chemical composition of the tested preparation was reported in only 3 of the 12 trials [17,19,42]. Accordingly, the evidence levels in Table 1 and Table 2 indicate which botanical ingredients have been clinically investigated, rather than establishing their efficacy.

### 3.4. General Characteristics of Study Population

A total of 13,519 DCFs were analyzed (female: *n* = 4451; male: *n* = 9068). The mean age of the athletes was 24.8 ± 5.7 years (females: 24.3 ± 5.4; males: 25.1 ± 5.8), and they represented 55 different sports disciplines. Doping controls were conducted both in-competition (*n* = 6785; 50.2%) and out-of-competition (*n* = 6734; 49.8%).

### 3.5. Patterns of Botanical Ingredient Declaration by Sex

Female athletes more frequently declared a single relevant botanical ingredient per DCF (4.16% of all DCFs, compared to 3.05% for male athletes), whereas male athletes more frequently declared two ingredients concurrently (1.67% compared to 1.10% for female athletes)—Table 3. The proportion of DCFs with exactly three relevant ingredients was similar for both sexes (0.18 and 0.19%, for females and males, respectively). Overall, at least one PMS- or dysmenorrhea-relevant ingredient was declared in 5.44% of DCFs among female athletes and 4.91% among male athletes. Among DCFs with at least one relevant ingredient, the distribution of ingredient counts differed significantly between sexes (χ^2^ = 14.9, *df* = 2, *p* < 0.001). As this comparison could not account for potential clustering of repeated observations from the same athlete, it is interpreted as exploratory.

### 3.6. Botanical Ingredients Identified in DCFs with Potential Relevance to PMS

**Figure 2:**
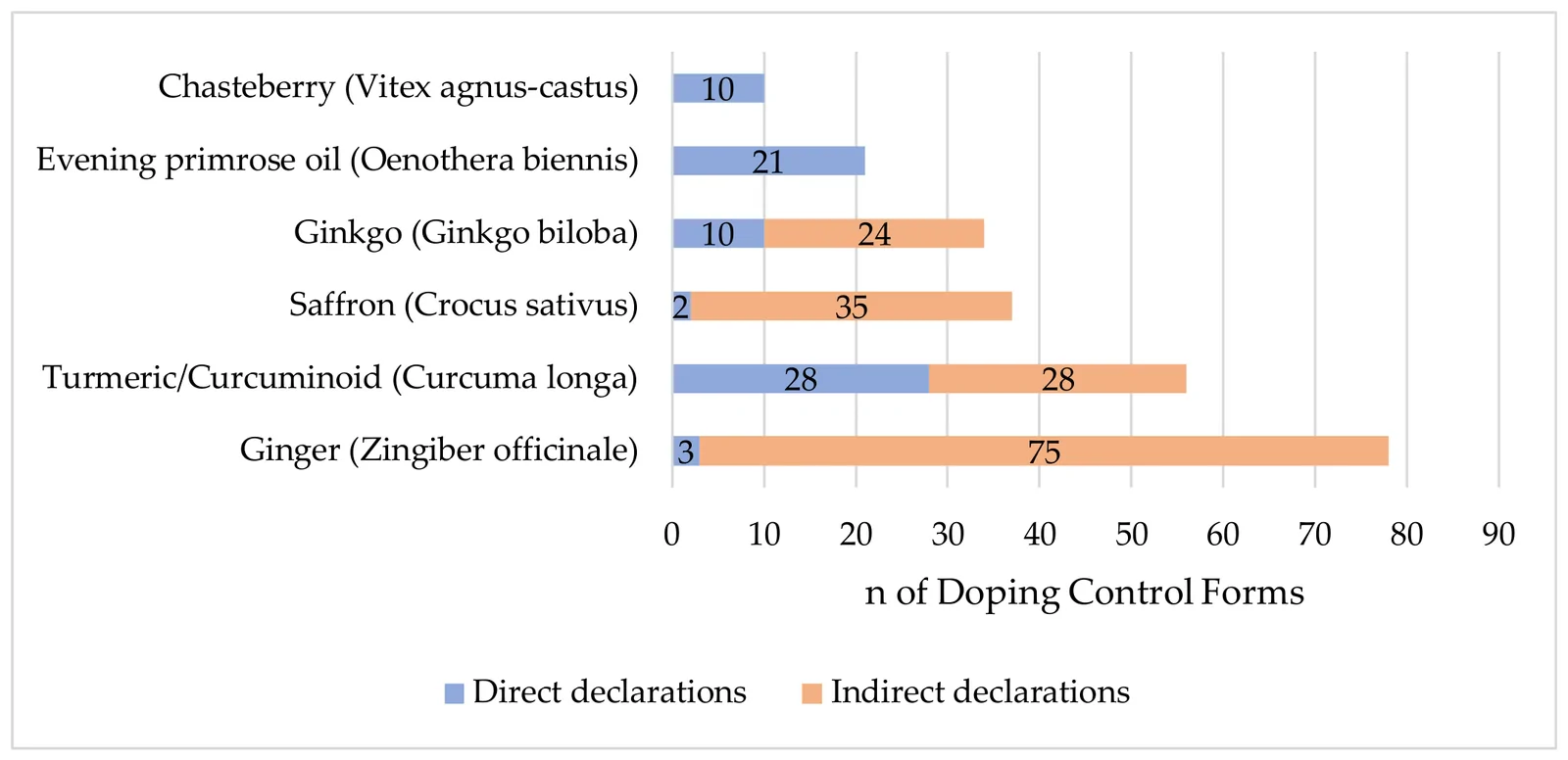
Botanical ingredients declared (both directly and indirectly) on Doping Control Forms by female athletes tested in Poland (2018–2023), which have the potential to alleviate symptoms of premenstrual syndrome.

Out of 4451 DCFs completed by female athletes, we identified six botanical ingredients with potential use in alleviating symptoms of PMS (Figure 2). The most frequently declared was ginger (*n* = 78), followed by turmeric/curcuminoid (*n* = 56), saffron (*n* = 37) and ginkgo (*n* = 34). Less frequently declared were evening primrose oil (*n* = 21) and chasteberry (*n* = 10).

### 3.7. Botanical Ingredients Identified in DCFs with Potential Relevance to Dysmenorrhea

**Figure 3:**
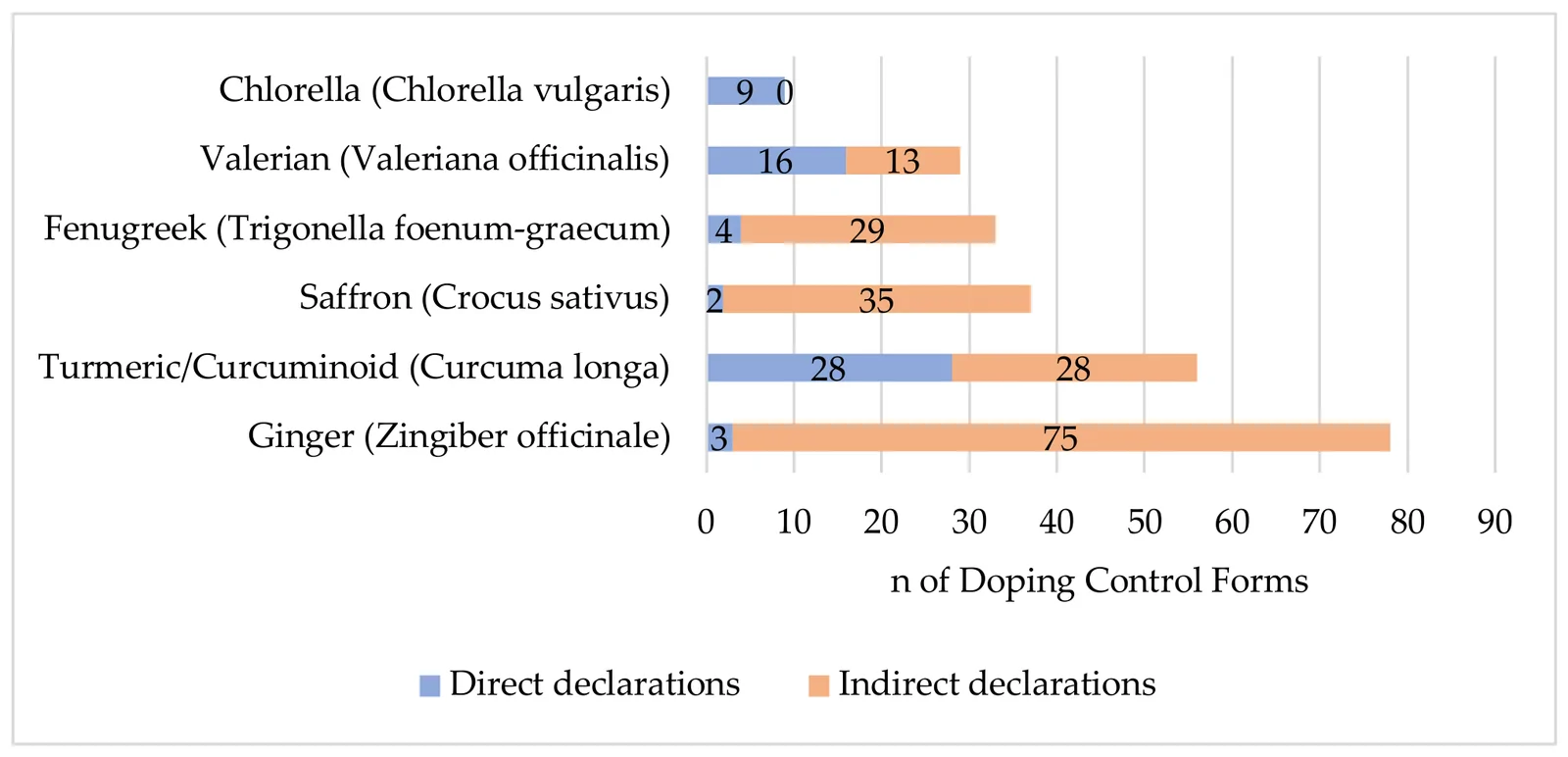
Botanical ingredients declared (both directly and indirectly) on Doping Control Forms by female athletes tested in Poland (2018–2023), which have the potential to alleviate symptoms of dysmenorrhea.

Six botanical ingredients that could potentially alleviate symptoms of dysmenorrhea were identified in the declarations (Figure 3). Ginger was the most frequently declared ingredient (*n* = 78) followed by turmeric/curcuminoid (*n* = 56) and saffron (*n* = 37). Fenugreek (*n* = 33), valerian (*n* = 29) and chlorella (*n* = 9) were declared less frequently.

### 3.8. Frequency of Botanical Ingredient Declaration in DCFs by Sex

Nine botanical ingredients identified in the narrative review were detected in the DCF dataset (Table 4), reflecting both directly declared ingredients and ingredients identified by the researchers from multi-ingredient product composition. After adjustment for multiple comparisons, statistically significant differences between female and male athletes in overall declaration rates were observed for five ingredients. Chasteberry (0.22% vs. 0.02%, adjusted *p* = 0.002), saffron (0.83% vs. 0.39%, adjusted *p* = 0.002), and evening primrose oil (0.47% vs. 0.03%, adjusted *p* < 0.001) were declared more frequently by female athletes. Fenugreek (0.74% vs. 1.78%, adjusted *p* < 0.001) and ginger (1.75% vs. 2.59%, adjusted *p* = 0.005) were declared more frequently by male athletes. No significant sex differences in total declaration rates were found for ginkgo, chlorella, valerian, or turmeric/curcuminoid.

To assess the sensitivity of the findings to uncertainty associated with indirect ingredient identification, sex-related comparisons were also examined using direct declarations only. These results are independent of the reconstruction of commercial product composition. Among directly declared ingredients, the overall proportion of DCFs naming an ingredient relevant to PMS or dysmenorrhea was significantly higher for female than male athletes (2.13% vs. 0.62% of all DCFs; χ^2^ = 62.2, *df* = 1, *p* < 0.001). At the individual substance level, significant sex differences in direct declaration were observed for evening primrose oil (0.47% vs. 0.03%, adjusted *p* < 0.001), valerian (0.36% vs. 0.03%, adjusted *p* < 0.001), chasteberry (0.22% vs. 0.02%, adjusted *p* = 0.002), and ginkgo (0.22% vs. 0.02%, adjusted *p* = 0.002), all of which were more frequently declared by female athletes. No significant sex differences in direct declarations were found for chlorella, ginger, fenugreek, saffron or turmeric/curcuminoid. These comparisons should be interpreted as exploratory, as clustering from potential repeated observations by the same athlete could not be excluded.

## 4. Discussion

Our narrative literature review identified a considerable number of botanical ingredients with reported clinical evidence for PMS (such as chasteberry, saffron and ginkgo), and dysmenorrhea (such as ginger, fennel and chamomile), suggesting that female athletes have a range of botanical options with reported clinical evidence available for managing these conditions. However, it should be noted that confidence in all appraised systematic reviews was low or critically low, and most individual trials were at high risk of bias. Patterns of botanical ingredients use differed between female and male athletes, with female athletes more frequently declaring a single relevant substance per DCF, whereas male athletes more often declared two ingredients concurrently. Among the botanical ingredients identified in the literature review as having potential efficacy for PMS and dysmenorrhea management, the most frequently declared by female athletes were ginger and turmeric/curcuminoid. Nevertheless, declarations of such botanical ingredients on DCFs were infrequent, appearing on no more than 1.75% of female-athlete DCFs.

The overall frequency of DCF declarations of botanical ingredients previously studied for PMS or dysmenorrhea management among female athletes was low. This finding should be interpreted with caution, as DCFs do not record symptom status, diagnosis, or the reason for use, and the proportion of athletes experiencing PMS or dysmenorrhea in this dataset is unknown. This low frequency can be considered in the context of the following observations: 1—elite athletes are generally willing to use dietary supplements [47,48], 2—plant-based treatment of PMS and dysmenorrhea has been reported to be more effective than placebo [7,8], and 3—reported PMS prevalence among athletes ranges widely, from 8.6% to 65.8% [1], indicating that a substantial proportion of this population could plausibly benefit from such management strategies. This raises the untested hypothesis, to be examined in future research, that athletes may be unaware of botanical options with supporting clinical evidence, such as chasteberry, or may be deliberately avoiding them for other reasons; however, this could not be determined in the present study. The use of any dietary supplement carries an inherent risk of inadvertent anti-doping rule violations, due to potential contamination with prohibited substances [49]. It may therefore be hypothesized that athletes avoid herbal supplements out of concern for anti-doping compliance; however, research indicates that awareness of contamination risks remains insufficient even among elite athletes [50,51]. Another possible explanation is that these athletes are relying on pharmacological treatment rather than botanical remedies. First-line pharmacological options for dysmenorrhea include nonsteroidal anti-inflammatory drugs (NSAIDs) and hormonal contraceptives [4], while PMS management similarly involves hormonal contraceptives and selective serotonin reuptake inhibitors (SSRIs) [52].

Given the low certainty of the reported clinical evidence for several botanical ingredients for PMS and dysmenorrhea management, the question of whether their use should be actively encouraged in athletic populations requires careful evaluation. Beyond anti-doping considerations, herbal supplements carry additional health risks that are often overlooked. The variable content of biologically active substances, undefined ingredients, and unclear labelling can pose direct health hazards [53]. Furthermore, ‘natural’ does not inherently mean safe: some botanical ingredients have been associated with hepatotoxicity, and interactions with medications commonly used by athletes have been documented. For example, oral contraceptives, used in the management of PMS, and St. John’s wort, used for its mood-modulating properties, when taken together can cause breakthrough bleeding [54].

The overall proportion of DCFs containing at least one PMS- or dysmenorrhea-relevant ingredient was similar for female and male athletes; however, the distribution of ingredient counts differed significantly: female athletes more often declared a single relevant ingredient, whereas male athletes more often declared two concurrently. Chasteberry and evening primrose oil showed the clearest female-predominant pattern, with several-fold higher declaration rates among female athletes and a comparatively small gap between total and directly declared use. Because the reason for use is not recorded on DCFs, this pattern cannot be interpreted as indicating use for menstrual-related symptoms; both ingredients are also used for other female-specific purposes [55]. The comparatively small gap between total and direct declarations for these two ingredients suggests that athletes were more often naming them directly rather than as incidental components of broader-purpose formulations. Valerian’s direct declaration rate was also significantly higher among female athletes. However, unlike with chasteberry and evening primrose oil, indirect declarations were an important indicator of valerian use in both sexes. The majority of valerian declarations among men were identified only through product composition rather than being named directly, and indirect declarations were particularly high in this group. Valerian is used both for sleep and anxiety [56] and, in some populations, specifically for dysmenorrhea-related pain and cramps [57]; therefore, its higher direct-declaration rate among female athletes may reflect either indication, and the data cannot distinguish between them.

Fenugreek and ginger showed a male-predominant pattern. The sports-science literature offers plausible explanations for this pattern that are unrelated to menstrual symptoms. Fenugreek has been studied primarily for its androgenic and anabolic properties [58] and ginger for its anti-inflammatory and analgesic effects in athletic populations [59]. Our analysis of the DCFs revealed that these botanical ingredients were declared by male athletes predominantly as components of products marketed as testosterone boosters and pre-workout supplements.

The most frequently declared botanical ingredients among female athletes with potential efficacy for PMS management were ginger and turmeric/curcuminoid, both classified as Level 2 in the evidence hierarchy. Research on turmeric, the second most popular supplement, is inconclusive. One study failed to show that it is more effective than a placebo [60]. However, other results suggest that a combination of curcuminoid and piperine can enhance radical scavenging activity [61] or decrease IgE levels [62] in young women with PMS and primary dysmenorrhea. Chasteberry, which had the most extensive (although low-confidence) evidence base for PMS management among the botanical ingredients reviewed, was among the least frequently declared supplements. Unlike piperine or ginger, which are also used for performance-related or general health purposes, chasteberry has no established ergogenic or broader health rationale outside of gynecological indications, making its low declaration rate among female athletes notable.

Among botanical ingredients identified for dysmenorrhea, ginger and turmeric/curcuminoid were declared most frequently in DCF by female athletes. However, ginger was also declared at significantly higher rates by male athletes. As an exploratory contextual comparison rather than a validated control for indication, this pattern may be more consistent with ginger’s broader use for exercise-related anti-inflammatory and analgesic purposes [59], than with a dysmenorrhea-specific explanation.

Several other substances, not included in our study, have been described in the literature as having potentially beneficial effects on the alleviation of PMS symptoms. Among them is roseroot, known commonly as *Rhodiola rosea*, which was not included in our review, as its clinical evidence for PMS did not meet our eligibility criteria. In the only placebo-controlled trial, roseroot was administered as one component of a multi-ingredient formulation, together with gamma-aminobutyric acid, chasteberry, vitamin B6 and melatonin, and no significant between-group differences versus placebo were observed [63]. Its independent effect therefore cannot be identified. A further study evaluated a polyherbal tea containing roseroot, fennel and lemon balm (*Melissa officinalis*), but used an uncontrolled single-arm design in 15 participants [64]. Nevertheless, roseroot appeared on 1.51% of DCFs completed by both female and male athletes. The high declaration frequency of roseroot is particularly noteworthy given the limited evidence supporting its efficacy and concerns regarding the quality of commercially available preparations. These concerns are substantiated by a recent investigation of 13 dietary supplements marketed in Poland and labelled as containing roseroot, which revealed that approximately 60% of the tested products did not meet their declared content specifications. Furthermore, the active compound rosavin was detected in only 9 of the 13 samples examined [65]. Roseroot is also commonly used in sport for other purposes, such as reducing post-exercise muscle pain and damage [66] and for its stress- and mood-related effects [67]. Another substance not included in this review was *Withania somnifera*, commonly known as Ashwagandha. Namysł et al. [68] proposed four distinct pathways through which Ashwagandha may be valuable in the management of PMS. These pathways encompass the modulation of the hypothalamic–pituitary–adrenal (HPA) axis, serotonergic activity, and anti-inflammatory properties. However, the authors emphasize that none of these mechanisms has been substantiated through clinical trials conducted specifically in PMS populations.

### 4.1. Strengths and Limitations

This study has several strengths. The use of DCFs enabled the examination of a large number of declarations from elite athletes, drawn from official anti-doping records maintained by the POLADA. This population is otherwise difficult to access for research purposes due to well-documented barriers to the participation of elite athletes in research [69]. Because DCFs are completed under standardized conditions as part of formal doping control procedures, this approach also minimizes recall bias compared to retrospective self-report surveys.

Our results should nonetheless be interpreted in light of some limitations. Firstly, because the reference set was defined by a targeted narrative search rather than a systematic review, some botanical ingredients with clinical evidence for PMS or dysmenorrhea may not have been identified, and ingredients not indexed under the general botanical terms may have been missed. Secondly, the trials summarized in Table 1 and Table 2 tested heterogeneous preparations, and only 3 of the 12 randomized controlled trials reported standardization or chemical composition (Table S4). As biological activity depends on composition, the reported effects cannot be generalized to all products containing the same botanical species. Moreover, although ingredients of declared products were identified from their labelled composition, many commercially available supplements do not report standardization; athletes’ preparations may therefore differ from those tested in clinical trials. In addition, DCFs do not include batch numbers or other information allowing the exact formulation used by an athlete to be identified; as product compositions were verified using sources available at the time of analysis, changes in product formulation over time cannot be excluded. Consequently, the indirect component of declaration counts represents a researcher-reconstructed classification: it depends on the investigators’ identification of each declared product and on composition sources that were not systematically archived, and is therefore not fully independently reproducible. Because indirect identification contributed substantially to the total declaration counts for several ingredients, the corresponding prevalence estimates and sex-related comparisons should be interpreted with caution. Thirdly, it should be acknowledged that the purpose for which athletes declared using the identified botanical ingredients cannot be determined from DCF data alone, as they are not recorded in standard DCF. Fourthly, DCF only considers the seven days preceding the doping control. Therefore, it is possible that some athletes may have previously used some of these botanical ingredients, but found them ineffective and subsequently discontinued their regular use. Additionally, because DCFs were fully anonymized, repeated observations from the same athlete could not be identified; any such clustering could have led the chi-squared comparisons to understate the true uncertainty of the reported *p*-values and 95% confidence intervals, which may therefore be too narrow, so between-sex comparisons should be interpreted as exploratory. Another limitation is that, in some DCFs, athletes did not specify the name of the supplement used and instead reported only the generic term “supplement.” Consequently, it was not possible to determine the exact product consumed. Therefore, it is plausible that some of these declarations referred to the botanical ingredients of interest in this study, suggesting that the frequency of their declaration may have been underestimated. Finally, potential confounding by sport discipline, age, or testing context could not be addressed because the number of declarations for individual botanical ingredients was too small for meaningful adjustment or stratification.

### 4.2. Practical Implications

Education on botanical options with reported clinical evidence, and on the anti-doping risks of supplement use, may be a reasonable broader recommendation; however, the present DCF data cannot establish a need for it. Further research is needed to examine the efficacy of botanical ingredients for PMS and dysmenorrhea management specifically in athletic populations, as evidence from this group remains scarce. Survey-based studies investigating motivations and frequency of botanical ingredient use among athletes would also provide valuable insight into the patterns observed in DCF data.

## 5. Conclusions

The declaration rates of botanical ingredients with reported clinical evidence for PMS and dysmenorrhea management were low among female athletes. Notably, some of the more frequently declared botanical ingredients were identified predominantly as components of multi-ingredient products, and the reason for their declaration cannot be determined from DCF data. Given that botanical products may vary in composition and quality, may carry a risk of contamination with prohibited substances, and may interact with medications, their use by athletes, particularly those subject to anti-doping regulations, should be undertaken with caution and under appropriate professional guidance.

## Figures and Tables

**Table 3:** Distribution of the number of premenstrual syndrome- and dysmenorrhea-relevant botanical ingredients declared per Doping Control Form (DCF), by sex.

Number of Botanical Ingredients per DCF	Females *n* = 4451		Males *n* = 9068	
	*n*	%	*n*	%
1	185	4.16	277	3.05
2	49	1.10	151	1.67
3	8	0.18	17	0.19
Any relevant botanical ingredient for PMS or dysmenorrhea	242	5.44	445	4.91

Chi-squared test comparing the distribution of the number of relevant botanical ingredients between sexes among DCFs with at least one relevant ingredient (conditional analysis): χ^2^ = 14.9, *df* = 2, *p* < 0.001. Independence between DCFs could not be verified; this comparison should be interpreted as exploratory. The number of DCFs without any relevant botanical ingredient was 4209 in female athletes and 8623 in male athletes.

**Table 4:** Declarations of premenstrual syndrome- and dysmenorrhea-relevant botanical ingredients among female and male athletes based on Doping Control Forms.

Botanical Ingredient	Number of Declarations	Females *n* = 4451		Males *n* = 9068		*p*-Value *	Adjusted *p*-Value **	PP (95% CI)	PR (95% CI)
		***n***	**%**	***n***	**%**				
Ginger (*Zingiber officinale*)	Total ^a^	78	1.75	235	2.59	0.002	0.005	−0.84 (−1.34; −0.33)	0.68 (0.52; 0.87)
	Direct ^b^	3	0.07	2	0.02	0.416	0.469	0.05 (−0.04; 0.13)	3.06 (0.51; 18.28)
Fenugreek (*Trigonella foenum-graecum*)	Total	33	0.74	161	1.78	<0.001	<0.001	−1.03 (−1.40; −0.66)	0.42 (0.29; 0.61)
	Direct	4	0.09	4	0.04	0.515	0.515	0.05 (−0.05; 0.14)	2.04 (0.51; 8.14)
Turmeric/Curcuminoid ^c^ (*Curcuma longa*)	Total	56	1.26	101	1.11	0.462	0.489	0.14 (−0.25; 0.54)	1.13 (0.82; 1.56)
	Direct	28	0.63	33	0.36	0.031	0.055	0.27 (0.00; 0.53)	1.73 (1.05; 2.86)
Ginkgo (*Ginkgo biloba*)	Total	34	0.76	46	0.51	0.068	0.101	0.26 (−0.04; 0.55)	1.51 (0.97; 2.34)
	Direct	10	0.22	2	0.02	<0.001	0.002	0.20 (0.06; 0.35)	10.19 (2.23; 46.47)
Saffron (*Crocus sativus*)	Total	37	0.83	35	0.39	<0.001	0.002	0.45 (0.15; 0.74)	2.15 (1.36; 3.41)
	Direct	2	0.04	0	0.00	0.205	0.264	0.04 (−0.02; 0.11)	Not estimable
Valerian (*Valeriana officinalis*)	Total	29	0.65	36	0.40	0.044	0.073	0.25 (−0.01; 0.52)	1.64 (1.01; 2.67)
	Direct	16	0.36	3	0.03	<0.001	<0.001	0.33 (0.15; 0.51)	10.87 (3.17; 37.27)
Evening primrose oil (*Oenothera biennis*)	Total	21	0.47	3	0.03	<0.001	<0.001	0.44 (0.23; 0.64)	14.26 (4.26; 47.79)
	Direct	21	0.47	3	0.03	<0.001	<0.001	0.44 (0.23; 0.64)	14.26 (4.26; 47.79)
Chlorella (*Chlorella vulgaris*)	Total	9	0.20	11	0.12	0.250	0.300	0.08 (−0.07; 0.23)	1.67 (0.69; 4.02)
	Direct	9	0.20	8	0.09	0.079	0.109	0.11 (−0.03; 0.26)	2.29 (0.88; 5.94)
Chasteberry (*Vitex agnus-castus*)	Total	10	0.22	2	0.02	<0.001	0.002	0.20 (0.06; 0.35)	10.19 (2.23; 46.47)
	Direct	10	0.22	2	0.02	<0.001	0.002	0.20 (0.06; 0.35)	10.19 (2.23; 46.47)

* Chi-squared test; Yates’ correction applied where expected cell count < 5; ** *p*-values adjusted for multiple comparisons using the Benjamini–Hochberg false discovery rate procedure across all 18 comparisons; independence between DCFs could not be verified; all comparisons in this table are exploratory. The confidence intervals are not adjusted for multiple comparisons. ^a^ refers to number of all DCFs in which specific botanical ingredient was identified, both directly (Direct) and as one of many ingredients of declared product (Indirect); ^b^ refers to number of DCFs in which specific botanical ingredient was directly stated; ^c^ includes declarations of turmeric and curcumin/curcuminoid-containing supplements, given their shared botanical source (*Curcuma longa*); CI—confidence interval; PP—percentage points; PR—prevalence ratio.

## Data Availability

The data analyzed in this study are derived from Doping Control Forms held by the Polish Anti-Doping Agency (POLADA) and were made available to the researchers solely for the purposes of this study. The underlying dataset cannot be made publicly available or shared by the authors because it contains confidential information collected as part of anti-doping activities and remains under the control of POLADA. The authors are not authorized to provide the dataset to third parties, and no controlled-access mechanism for external researchers is available. The corresponding author may be contacted for reasonable requests for additional information regarding the study methodology or aggregated results, but not for access to the underlying individual-level data.

## Supplementary Materials

The following supporting information can be downloaded at: https://www.mdpi.com/article/10.3390/nu18203455/s1, Table S1: Search terms used to guide the narrative literature search on botanical ingredients for premenstrual syndrome and dysmenorrhea; Table S2: Item-level quality assessment of systematic reviews and meta-analyses of botanical ingredients for premenstrual syndrome and dysmenorrhea included in the narrative review, based on AMSTAR 2; Table S3: Details of the risk of bias assessment (RoB 2) for randomized controlled trials evaluating botanical ingredients for premenstrual syndrome (PMS) and dysmenorrhea; Table S4: Characteristics of randomized controlled trials evaluating botanical ingredients for premenstrual syndrome and dysmenorrhea.

## Abbreviations

The following abbreviations are used in this manuscript:

DCF	Doping Control Form
HPA	Hypothalamic–pituitary–adrenal
NSAID	Nonsteroidal anti-inflammatory drugs
OCEBM	Oxford Centre for Evidence-Based Medicine
POLADA	Polish Anti-Doping Agency
PMS	Premenstrual syndrome
SSRI	Selective serotonin reuptake inhibitors
WJHXD	Wenjing Huoxue Decoction
