# Supplementary material for: Use of Botanical Ingredients Relevant to Premenstrual Symptoms and Dysmenorrhea Among Elite Female Athletes: Evidence from Doping Control Forms
Source: Nutrients. 2026 Oct 21;18(20):3455. doi: 10.3390/nu18203455 (PMC13708608; doi:10.3390/nu18203455)
Supplement: Supplementary file 1 [file nutrients-18-03455-s001.zip › nutrients-4571382-supplementary.pdf]

Table S1. Search terms used to guide the narrative literature search on botanical ingredients for premenstrual syndrome and dysmenorrhea.

Search	Search strategy
General search	("PMS" OR "PMDD" OR "premenstrual syndrome" OR "premenstrual dysphoric disorder" OR "premenstrual tension" OR "premenstrual symptoms" OR "dysmenorrhea" OR "dysmenorrhoea" OR "primary dysmenorrhea" OR "menstrual pain" OR "painful menstruation" OR "menstrual cramp" OR "menstrual cramps" OR "period pain") AND (herbal OR botanical OR botanicals OR phytotherap* OR "herbal medicine" OR "herbal extract" OR "herbal supplement" OR "herbal supplements" OR "plant extract" OR "plant extracts" OR "medicinal plant" OR "medicinal plants" OR "traditional Chinese medicine" OR "Chinese herbal medicine" OR nutraceutical*) AND ("randomized controlled trial" OR "randomised controlled trial" OR "systematic review" OR "meta-analysis" OR "clinical trial")
Premenstrual syndrome	("PMS" OR "PMDD" OR "premenstrual syndrome" OR "premenstrual dysphoric disorder" OR "premenstrual tension" OR "premenstrual symptom") AND ("chasteberry" OR "chaste tree" OR "Vitex agnus-castus" OR "saffron" OR "Crocus sativus" OR "ginkgo" OR "Ginkgo biloba" OR "evening primrose oil" OR "Oenothera biennis" OR "Oleum oenotherae" OR "turmeric" OR "Curcuma longa" OR "curcumin" OR "curcuminoid*" OR "roseroot" OR "Rhodiola rosea" OR "St. John's wort" OR "Hypericum perforatum" OR "wild yam" OR "Dioscorea" OR "ginger" OR "Zingiber officinale") AND ("randomized controlled trial" OR "randomised controlled trial" OR "systematic review" OR "meta-analysis" OR "clinical trial")
Dysmenorrhea	("dysmenorrhea" OR "dysmenorrhoea" OR "primary dysmenorrhea" OR "menstrual pain" OR "painful menstruation" OR "menstrual cramp*" OR "period pain") AND ("ginger" OR "Zingiber officinale" OR "fennel" OR "Foeniculum vulgare" OR "chamomile" OR "Matricaria chamomilla" OR "Matricaria recutita" OR "Damask rose" OR "Rosa damascena" OR "cinnamon" OR "Cinnamomum cassia" OR "dill" OR "Anethum graveolens" OR "saffron" OR "Crocus sativus" OR "Wenjing Huoxue" OR "Wen Jing Huo Xue" OR "Corydalis yanhusuo" OR "Glycyrrhiza glabra" OR "Commiphora myrrha" OR "Shaofu Zhuyu" OR "Shao Fu Zhu Yu" OR "Danggui Shaoyao San" OR "Dang Gui Shao Yao San" OR "Xuefu Zhuyu" OR "Xue Fu Zhu Yu" OR "guava" OR "Psidium guajava" OR "rhubarb" OR "Rheum emodi" OR "Rheum australe" OR "wild yam" OR "Dioscorea" OR "chlorella" OR "Chlorella vulgaris" OR "Chlorella pyrenoidosa" OR "turmeric" OR "Curcuma longa" OR "curcumin" OR "curcuminoid*" OR "fenugreek" OR "Trigonella foenum-graecum" OR "valerian" OR "Valeriana officinalis" OR "Zataria multiflora" OR "zataria" OR "dried fig*" OR "Ficus carica" OR "Stachys lavandulifolia") AND ("randomized controlled trial" OR "randomised controlled trial" OR "systematic review" OR "meta-analysis" OR "clinical trial")

Table S2. Item-level quality assessment of systematic reviews and meta-analyses of botanical ingredients for premenstrual syndrome and dysmenorrhea included in the narrative review, based on AMSTAR 2

Ref	Botanical ingredients appraised	1	2*	3	4*	5	6	7*	8	9*	10	11*	12	13*	14	15*	16	Critical flaws (items)	Overall confidence
[23]	Chasteberry	Y	Y	Y	PY	Y	Y	PY	Y	Y	N	Y	N	Y	Y	N	Y	15	Low
[24]	Chasteberry	Y	N	Y	Y	Y	Y	PY	Y	Y	N	Y	Y	Y	Y	Y	Y	2	Low
[25]	Chasteberry, saffron, ginkgo	PY	N	Y	PY	N	N	Y	Y	N	N	NA	NA	Y	Y	NA	Y	2, 9	Critically low
[26]	Saffron	Y	N	N	Y	Y	Y	Y	Y	Y	N	Y	N	N	Y	Y	Y	2, 13	Critically low
[32]	Ginger, fennel, cinnamon	Y	N	Y	PY	N	Y	PY	Y	PY	N	Y	Y	N	Y	Y	Y	2, 13	Critically low
[33]	Ginger	Y	PY	N	PY	Y	Y	N	Y	N	N	N	N	Y	Y	N	Y	7, 9, 11, 15	Critically low
[34]	Ginger, chamomile, guava leaf, rhubarb, fenugreek, valerian, zataria	Y	Y	Y	Y	Y	Y	Y	Y	Y	N	Y	N	Y	Y	N	Y	15	Low
[35]	Fennel, dill	Y	Y	Y	Y	Y	N	N	PY	Y	N	Y	N	Y	Y	N	Y	7, 15	Critically low
[36]	Chamomile	Y	N	Y	Y	Y	N	PY	Y	Y	N	NA	NA	N	Y	NA	Y	2, 13	Critically low
[37]	Damask rose	Y	Y	Y	Y	Y	Y	Y	Y	Y	N	Y	N	N	Y	Y	Y	13	Low
[38]	Wenjing Huoxue Decoction	Y	Y	Y	Y	N	Y	PY	Y	Y	N	Y	N	Y	Y	N	Y	15	Low
[39]	Shaofu Zhuyu decoction	Y	Y	Y	Y	Y	Y	N	Y	Y	N	Y	N	Y	Y	N	Y	7, 15	Critically low
[40]	Danggui Shaoyao San	Y	Y	Y	Y	Y	Y	N	Y	Y	N	Y	N	Y	Y	N	Y	7, 15	Critically low
[41]	Xuefu Zhuyu decoction	Y	Y	Y	Y	Y	Y	PY	Y	Y	N	Y	N	Y	Y	N	Y	15	Low

Y – yes; PY – partial yes; N – no; NA – not applicable (no meta-analysis conducted; items 11, 12 and 15). * Critical domain; a critical flaw is an N in a critical domain. Overall confidence was rated as high (no or one non-critical weakness), moderate (more than one non-critical weakness), low (one critical flaw, with or without non-critical weaknesses) or critically low (more than one critical flaw, with or without non-critical weaknesses); no summary score was calculated. **AMSTAR 2 items:** (1) PICO components in research question and inclusion criteria; (2) Review methods established a priori (protocol) and deviations justified; (3) Explanation of selection of study designs for inclusion; (4) Comprehensive literature search strategy; (5) Study selection performed in duplicate; (6) Data extraction performed in duplicate; (7) List of excluded studies with justification provided; (8) Included studies described in adequate detail; (9) Satisfactory technique for assessing risk of bias in individual studies; (10) Sources of funding for included studies reported; (11) Appropriate methods for statistical combination of results; (12) Assessment of potential impact of risk of bias in individual studies on meta-analysis results; (13) Risk of bias in individual studies accounted for when interpreting/discussing results; (14) Satisfactory explanation for, and discussion of, heterogeneity; (15) Adequate investigation of publication bias and discussion of its likely impact; (16) Conflicts of interest reported, including funding received for the review.

Table S3. Details of the risk of bias assessment (RoB 2) for randomized controlled trials evaluating botanical ingredients for premenstrual syndrome (PMS) and dysmenorrhea

Botanical ingredient	Reference	Design	Outcome	Time point	Comparison	D1	D2	D3	D4	D5	DS	Overall
**Premenstrual syndrome**												
Evening primrose oil (*Oenothera biennis*)	[27][a]	Parallel	TDSR	End of cycle 2	Femicomfort vs. placebo	Low	Low	Low	Low	Some concerns	NA	Some concerns
	[28]	Parallel	Total symptom severity score	3 months	EPO vs. placebo	Some concerns	High	High	Low	Some concerns	NA	High
Curcumin (*Curcuma longa*)	[29]	Parallel	Total PMS score	End of cycle 3	Curcumin vs. placebo	Low	Low	Low	Low	Some concerns	NA	Some concerns
St. John's wort (*Hypericum perforatum*)	[17]	Crossover	DSR total and subscale scores, within-participant comparison	End of each 2-cycle treatment period	St. John's wort tablets vs. placebo	Low	Low	Low	Low	Low	Low	Low
Wild yam (*Dioscorea esculenta*)	[18]	Crossover	DRSP; menstrual pain (VAS, MPQ), within-participant comparison	Day 2 of menses after each 4-week treatment period	Tuber powder vs. placebo	Some concerns	Some concerns	Low	Some concerns	High	Some concerns	High
Ginger (*Zingiber officinale)*	[30]	Parallel	Total DSR score	End of cycle 3	Ginger vs. placebo	Some concerns	High	Some concerns	Low	Some concerns	NA	High
**Dysmenorrhea**												
Guava leaf (*Psidium guajava*)	[42]	Parallel	VAS	End of cycle 3	Extract vs. placebo[b]	Some concerns	High	Some concerns	Low	Some concerns	NA	High
Rhubarb (*Rheum emodi*)	[43]	Parallel	VAS	End of cycle 3	Rhubarb vs. mefenamic acid	High	Some concerns	Low	Some concerns	Low	NA	High
Chlorella (*Chlorella vulgaris*)	[44]	Parallel	VAS	Week 8	Chlorella vs. placebo	Low	Low	Low	Low	Low	NA	Low
Turmeric (*Curcuma longa*)	[45]	Parallel	VAS	End of intervention	Turmeric vs. placebo[b]	Some concerns	Low	Low	Low	Low	NA	Some concerns
Dried figs (*Ficus carica*)	[46]	Parallel	VAS	Cycle 3	Dried figs vs. placebo[b]	Some concerns	High	High	High	Some concerns	NA	High

Stachys lavandulifolia (*Stachys lavandulifolia*)	[19]	Crossover	VAS	Day 4 of treatment cycle	Stachys extract vs. placebo	High	Low	Low	Low	Low	Low	High

*D1 – randomization process; D2 – deviations from intended interventions; D3 – missing outcome data; D4 – measurement of the outcome; D5 – selection of the reported result; DS – bias arising from period and carryover effects (crossover trials only). In all trials, the effect of interest was the effect of assignment to the intervention (intention-to-treat). Parallel-group trials were assessed with the standard RoB 2 tool[16]; crossover trials were assessed with the RoB 2 version for crossover trials, including Domain S;

[a] Multi-ingredient formulation: Femicomfort (evening primrose oil, vitamin B6 and vitamin E); the independent effect of the botanical cannot be identified. [b] Trial included additional arms (guava: second dose and ibuprofen; turmeric: mefenamic acid and turmeric plus mefenamic acid; figs: cinnamon); only the comparison with placebo was assessed. DRSP – Daily Record of Severity of Problems, DSR - Daily Symptom Report, EPO – evening primrose oil, TDSR – Total Daily Symptom Report score, VAS -visual analogue scale

Table S4. Characteristics of randomized controlled trials evaluating botanical ingredients for premenstrual syndrome and dysmenorrhea

Botanical ingredient	Reference	Study design (n)	Single-vs. multi-ingredient (SI/MI)	Plant part / extract type	Standardization and key constituents	Formulation	Dose and duration	Comparator	Overall risk of bias (RoB2)
**Premenstrual syndrome**									
Evening primrose oil (*Oenothera biennis*)	[27]	DB, RCT (40)	MI (evening primrose oil, vitamin B6, vitamin E); effect of evening primrose oil cannot be isolated	NR / NR	NR	Capsule	NR / 2 MC	Placebo	Some concerns
	[28]	TB, RCT (80)	SI	NR / NR	NR	Capsule	1500 mg/day, 3 months	Placebo	High
Curcumin *(Curcuma longa)*	[29]	DB, RCT (70)	SI	Isolated curcumin powder	NR	Capsule	200 mg/day, from 7 days before to 3 days after menses onset, for 3 consecutive cycles	Placebo	Some concerns
St. John's wort (*Hypericum perforatum*)	[17]	DB, RCT (36)	SI	Extract Li160, 80% methanolic dry extract, drug-to-extract ratio 3–6:1	0.18% hypericin, 3.38% hyperforin	Coated tablet	900 mg/day (2x450 mg), 2 MC	Placebo	Low
Wild yam (*Dioscorea esculenta*)	[18]	DB, RCT (10)	SI	Tuber powder (source of diosgenin)	NR	Capsule	300 mg/day, 4 weeks	Placebo	High

Ginger (*Zingiber officinale)*	[30]	DB, RCT (70)	SI	NR	NR	Capsule	500 mg/day (2x250 mg), from 7 days before to 3 days after menses onset, 3 MC	Placebo	High
**Dysmenorrhea**									
Guava leaf (*Psidium guajava*)	[42]	DB, RCT (197)	SI	Leaf (folium), ethanolic extract	Standardized flavonol content, 1 mg active ingredient per 300 mg extract	Capsule	3 mg/day or 6 mg/day, for 5 days per cycle (24h before menses to end of bleeding), 3 MC	Placebo and ibuprofen	High
Rhubarb (*Rheum emodi*)	[43]	Single-blind, RCT (45)	SI	Dried root/rhizome, powdered	NR	Capsule (~420 mg powdered drug per 500 mg capsule)	3 capsules twice daily (≈2.5 g/day), from 2 days before menses through first 3 days of menstruation, 3 MC	Mefenamic acid 250 mg	High
Chlorella (*Chlorella vulgaris*)	[44]	DB, RCT (44)	SI	Whole-organism supplement (unicellular green algae)	NR	Soft gel capsule	1500 mg/day, 8 weeks	Placebo	Low
Turmeric (*Curcuma longa*)	[45]	DB, RCT (128)	SI (turmeric-only arm); a separate arm received	Rhizome, powdered (not an extract)	NR	Capsule	500 mg/day, (2 days before to 3 days after	Placebo and mefenamic acid (250 mg) and	Some concerns

			turmeric plus mefenamic acid and was not used				menses onset); duration NR	turmeric+mefenamic combination	
Dried figs (*Ficus carica*)	[46]	Randomized, unblinded (107)	SI	Whole dried fruit (not an extract)	Not applicable	Whole fig	6 dried figs/day, days 1–3 of 3 MC	Cinnamon powder (active comparator, also unblinded) and placebo (empty capsule)	High
Stachys lavandulifolia (*Stachys lavandulifolia*)	[19]	DB, RCT (29)	SI; co-intervention: mefenamic acid permitted as rescue analgesic in all cycles	Aerial parts, hydroalcoholic extract	Standardized to 0.15 mg thymol per 100 mg extract per capsule	Capsule	Dose/frequency not explicitly stated as mg/day (taken "every 4–6h" for 4 days: 1 day before to 3 days after menses onset), 3 MC	Placebo	High

DB – double blind, MI -multi-ingredient intervention, TB – triple blind, RCT – randomized controlled trial, SI – single ingredient intervention, NR – not reported, MC – menstrual cycle,
